# Primary Pyogenic Ventriculitis in Adults: A Retrospective Case Series of Clinical Characteristics, Cerebrospinal Fluid Parameters, and Surgical Drain Managements

**DOI:** 10.3390/jcm15207992

**Published:** 2026-10-15

**Authors:** Yi-Jen Ho, Hsiang-Ling Huang, Po-Kai Wang, Sheng-Tzung Tsai

**Affiliations:** 1 Department of Neurosurgery, Hualien Tzu Chi Hospital, Buddhist Tzu Chi Medical Foundation, Hualien 970, Taiwan; 114330108@gms.tcu.edu.tw (Y.-J.H.); 106321110@gms.tcu.edu.tw (H.-L.H.); 2 Institute of Medical Sciences, Tzu Chi University, Hualien 970, Taiwan; 3 Department of Nursing, Hualien Tzu Chi Hospital, Buddhist Tzu Chi Medical Foundation, Hualien 970, Taiwan; 4 Department of Anesthesiology, Hualien Tzu Chi Hospital, Buddhist Tzu Chi Medical Foundation, Hualien 970, Taiwan; pk661113@gmail.com; 5 School of Medicine, Tzu Chi University, Hualien 970, Taiwan

**Keywords:** primary pyogenic ventriculitis, diffusion-weighted imaging, cerebrospinal fluid, external ventricular drain, central nervous system infection, restricted diffusion

## Abstract

**Background/Objectives:** Primary pyogenic ventriculitis in adults is a rare, life-threatening central nervous system infection occurring in the absence of prior neurosurgical interventions or indwelling medical devices. Due to its scarcity, individual clinical trajectories, cerebrospinal fluid (CSF) cytochemical variations, and external ventricular drain (EVD) management timing remain poorly defined. **Methods:** We conducted a retrospective case series of *N* = 8 with confirmed primary pyogenic ventriculitis admitted to a tertiary referral center in eastern Taiwan between 2019 and 2023. Individual-level clinical, neuroimaging, CSF, and surgical timing data were analyzed descriptively. **Results:** The cohort was 62.5% male (*n* = 5/8) with a median age of 60.5 years [IQR: 51.5–66.5]. All patients presented with severe initial neurological impairment (median initial Glasgow Coma Scale [GCS] = 7.5 [IQR: 4.0–9.75]). Overall 1-year mortality was 25.0% (*n* = 2/8, both male). Survivors (*n* = 6) exhibited marked initial CSF pleocytosis (median WBC = 1005.0 cells/µL [IQR: 508.0–1977.5]) and elevated protein (median = 1030.0 mg/dL [IQR: 847.5–1225.0]). Conversely, non-survivors (*n* = 2) demonstrated blunted initial CSF inflammatory markers (median WBC = 82.5 cells/µL; median protein = 212.1 mg/dL) alongside severe initial coma (median GCS = 3.5) and systemic shock requiring vasopressors. EVD placement occurred at a median of 2.5 days post-onset in non-survivors compared to 10.5 days in survivors, reflecting urgent, reactive surgical intervention in rapidly deteriorating cases (confounding by indication). At 1 year, surviving patients achieved a median modified Rankin Scale (mRS) score of 2.5 [IQR: 2.0–3.0]. **Conclusions:** In this rare adult primary pyogenic ventriculitis series, fatal cases were characterized by severe initial coma, vasopressor-dependent shock, and blunted CSF cellularity. Urgent EVD placement in non-survivors represented reactive rescue therapy. Future prospective multicenter registries are required to evaluate whether standardized, early intraventricular source control improves functional outcomes.

## 1. Introduction

Primary pyogenic ventriculitis in adult is an exceptionally rare, fulminant central nervous system (CNS) infection characterized by suppurative inflammation of the ependymal lining of the cerebral ventricles [1,2]. The vast majority of contemporary neurosurgical and neurocritical care literature focuses on healthcare-associated ventriculitis (HAV), which typically occurs as a nosocomial complication secondary to neurosurgical interventions, penetrating head trauma, or indwelling intracranial devices such as external ventricular drains (EVDs) and ventriculoperitoneal shunts [1]. In contrast, primary ventriculitis emerges spontaneously in patients without prior neurosurgical exposure, arising either via hematogenous dissemination of community-acquired pathogens or as a secondary extension from severe bacterial meningitis or ruptured brain abscesses [3,4].

The epidemiological and clinical burden of primary ventriculitis is substantial. In a recent multicenter retrospective cohort study of 87 ventriculitis patients by Díez-Vidal et al., a 1-year all-cause mortality rate of 39.1% was reported, with 67.3% of discharged survivors suffering from long-term neurological sequelae. However, this cohort comprised predominantly healthcare-associated and post-surgical device-related ventriculitis cases rather than primary pyogenic infections. Because device-related ventriculitis involves distinct catheter-associated biofilm dynamics, nosocomial pathogen profiles, and surgical drain retention protocols, direct extrapolation of these mortality figures to spontaneous primary pyogenic ventriculitis requires careful contextualization [3,5].

Pathophysiologically, circulating pathogens gain entrance into the ventricular system by exploiting the highly vascularized choroid plexus, which lacks the continuous tight junctions characteristic of the traditional blood–brain barrier (BBB) [5]. Once inoculated inside the ventricular system, bacteria proliferation triggers an intense local inflammatory cascade, promoting the accumulation of high-viscosity, protein-rich purulent exudates, ependymal denudation and intraventricular biofilm formation [6]. Among community-acquired pathogens, species such as *Streptococcus pneumoniae* and *Neisseria meningitidis* can induce rapid ependymal necrosis, presenting with subtle or atypical clinical features that frequently delay recognition until catastrophic brainstem compression occurs [4,7].

Establishing a timely microbiological diagnosis in primary pyogenic ventriculitis remains an acute clinical hurdle. Because empirical systemic antimicrobial therapy is almost universally initiated at primary referring facilities prior to tertiary neurosurgical transfer, cerebrospinal fluid (CSF) culture positivity rates are notoriously low, ranging from 20% to 30% [7,8]. Furthermore, systemic intravenous antimicrobials struggle to achieve effective therapeutic minimum inhibitory concentrations (MIC) within the purulent ventricular compartments due to restricted BBB/blood-CSF barrier penetration and active efflux pump [9]. Consequently, modern diagnostic protocols rely heavily on neuroimaging adjuvants. High-resolution magnetic resonance imaging (MRI)—specifically Diffusion-Weighted Imaging (DWI) paired with Apparent Diffusion Coefficient (ADC) maps and contrast-enhanced T1-weighted sequences—has become an indispensable diagnostic tool by demonstrating restricted water diffusion of purulent ventricular debris and smooth linear ependymal enhancement [10,11]. To overcome low culture yields, molecular diagnostics such as CSF metagenomic next-generation sequencing (mNGS) are also emerging as promising tools for rapid pathogen identification [2].

Despite these diagnostic advances, evaluating rare neuroinfectious entities like primary ventriculitis remains methodologically challenging. Because individual centers encounter only sporadic cases (*n* < 10), traditional inferential statistical modeling often lacks sufficient statistical power and is highly sensitive to extreme clinical outliers. To advance clinical understanding without over-extrapolating preliminary data, there is a clear consensus on the need for transparent, unmasked, individual-level case series that strictly adhere to international reporting standards (such as the CARE guidelines).

To address these critical clinical and methodological gaps, we present a retrospective case series of *N* = 8 adult patients with primary pyogenic ventriculitis admitted to a principal tertiary referral center in eastern Taiwan over a 5-year period (2019–2023). Adhering strictly to the CAse REport (CARE) guidelines, this study provides complete, unmasked, per-patient clinical, neuroimaging, CSF cytochemical, and surgical management data. By descriptively evaluating these rare cases alongside systemic confounders and the clinical context of EVD placement timing, we aim to delineate the real-world heterogeneity of adult primary ventriculitis and offer modest, hypothesis-generating insights to guide future prospective registries and early surgical protocols.

## 2. Material and Methods

### 2.1. Study Design and Ethical Approval

This study was conducted at Hualien Tzu Chi Hospital, the sole tertiary referral medical center serving eastern Taiwan—a geographically expansive region characterized by mountainous terrain, variable travel times to emergency care, and a demographically diverse population including Taiwanese Indigenous communities. Due to the retrospective observational design utilizing electronic health records, detailed individual parameters regarding socioeconomic status, exact indigenous ancestry, rural versus urban residence, and historical vaccination status were not systematically documented and could not be evaluated. This study was conducted as a single-center retrospective case series following the CAse REport (CARE) guidelines. The study protocol was approved by the Institutional Review Board (IRB) of Hualien Tzu Chi Hospital, Buddhist Tzu Chi Medical Foundation (IRB No.: IRB115-194-B; approved on 1 September 2026). Owing to the retrospective observational nature of the study using anonymized medical records, the requirement for individual patient informed consent was waived by the IRB, in full compliance with the Declaration of Helsinki.

### 2.2. Patient Eligibility and Diagnostic Criteria

We systematically screened the electronic medical records of all adult patients (≥18 years) admitted to the Department of Neurosurgery or Neuro-ICU with ICD-10 codes corresponding to intracranial abscess, ventriculitis, or pyogenic meningitis between 1 January 2019, and 31 December 2023. To strictly enforce the boundary of primary pyogenic ventriculitis, patients were included only if they met all of the following criteria: (1) a clinical presentation consistent with acute severe CNS infection, including fever, meningismus, altered consciousness, or focal neurological deficits; (2) neuroimaging confirmation on MRI demonstrating characteristic ependymal contrast enhancement along the ventricular walls, intraventricular purulent debris with restricted diffusion on Diffusion-Weighted Imaging (DWI), or acute obstructive hydrocephalus secondary to ventricular debris; (3) a confirmed purulent or inflammatory CSF profile characterized by an elevated WBC count, elevated protein, or depressed glucose; and (4) the complete absence of prior neurosurgical procedures, penetrating head trauma, or indwelling intracranial devices (such as external ventricular drains [EVDs], Ommaya reservoirs, or ventriculoperitoneal shunts) prior to presentation. Immunocompetence was explicitly defined as the absence of active immunosuppressive or cytotoxic therapy, solid organ or hematological malignancy, human immunodeficiency virus (HIV) infection, chemotherapy, or active systemic autoimmune disorders. Chronic metabolic conditions, including diabetes mellitus and systemic hypertension, were categorized as underlying vascular and metabolic comorbidities rather than primary immunodeficiencies. For culture-negative cases, diagnostic eligibility was strictly established through the combination of acute purulent CSF cytochemical profiles, classic clinical meningeal signs, characteristic intraventricular restricted diffusion on DWI/ADC MRI, and rigorous clinical exclusion of secondary or iatrogenic infectious pathways.

### 2.3. Data Collection and Variable Definitions

Individual patient data were independently extracted from electronic health records using a standardized data extraction template. Extracted parameters encompassed demographic and comorbidity profiles (age, sex, systemic hypertension, diabetes mellitus, chronic kidney disease, alcoholism, liver cirrhosis, and coronary artery disease, alongside admission systemic blood glucose levels to evaluate metabolic confounding on CSF glucose), clinical severity metrics (Glasgow Coma Scale score at presentation, presence of septic shock, and continuous vasopressor requirement upon ICU admission), and microbiological evaluation results (aerobic, anaerobic, fungal, and mycobacterial cultures from blood and CSF). Furthermore, neuroimaging features evaluated by a senior neuroradiologist were documented, specifically recording intraventricular DWI restricted diffusion of debris, ependymal contrast enhancement, and acute hydrocephalus. CSF sampling context parameters included the exact collection source (lumbar puncture vs. external ventricular drain [EVD]), sampling timing (hospitalization day post-symptom onset), initial WBC count, total protein concentration, and glucose concentration. Surgical drain management variables encompassed indication, technique, and the exact post-symptom onset day of passive EVD placement. Finally, clinical outcomes evaluated included 1-year all-cause mortality and functional disability measured via the modified Rankin Scale (mRS) score, where mRS 0–3 indicated favorable to moderate functional independence and mRS 6 indicated death.

### 2.4. Statistical Analysis and Data Presentation

In accordance with CARE guidelines and methodological recommendations for rare disease series (*N* = 8), no inferential hypothesis testing or comparative *p*-value calculations were performed. Reporting group comparisons (e.g., n1 = 6 survivors vs. n2 = 2 non-survivors) via asymptotic normal approximations or non-parametric tests yields invalid statistical inference and circular reasoning when evaluating predetermined mortality outcomes.

All quantitative continuous variables are presented purely as descriptive non-parametric summaries using the Median and Interquartile Range (IQR: 25th–75th percentiles) alongside absolute ranges where appropriate. Categorical variables are expressed as absolute counts and percentages (*n*/*N*, %). To ensure complete diagnostic transparency and data availability, all clinical, imaging, CSF, and outcome variables are fully tabulated at the individual patient level in an unmasked master data table (Table 1). Statistical data formatting was performed using Python (v3.12, Python Software Foundation, Wilmington, DE, USA).

During the preparation of this manuscript, the authors used Gemini (Google, Mountain View, CA, USA) for the purpose of academic language editing and proofreading. The authors have reviewed and edited the output and take full responsibility for the content of this publication.

## 3. Results

### 3.1. Baseline Characteristics and Clinical Presentation

**Figure 1:**
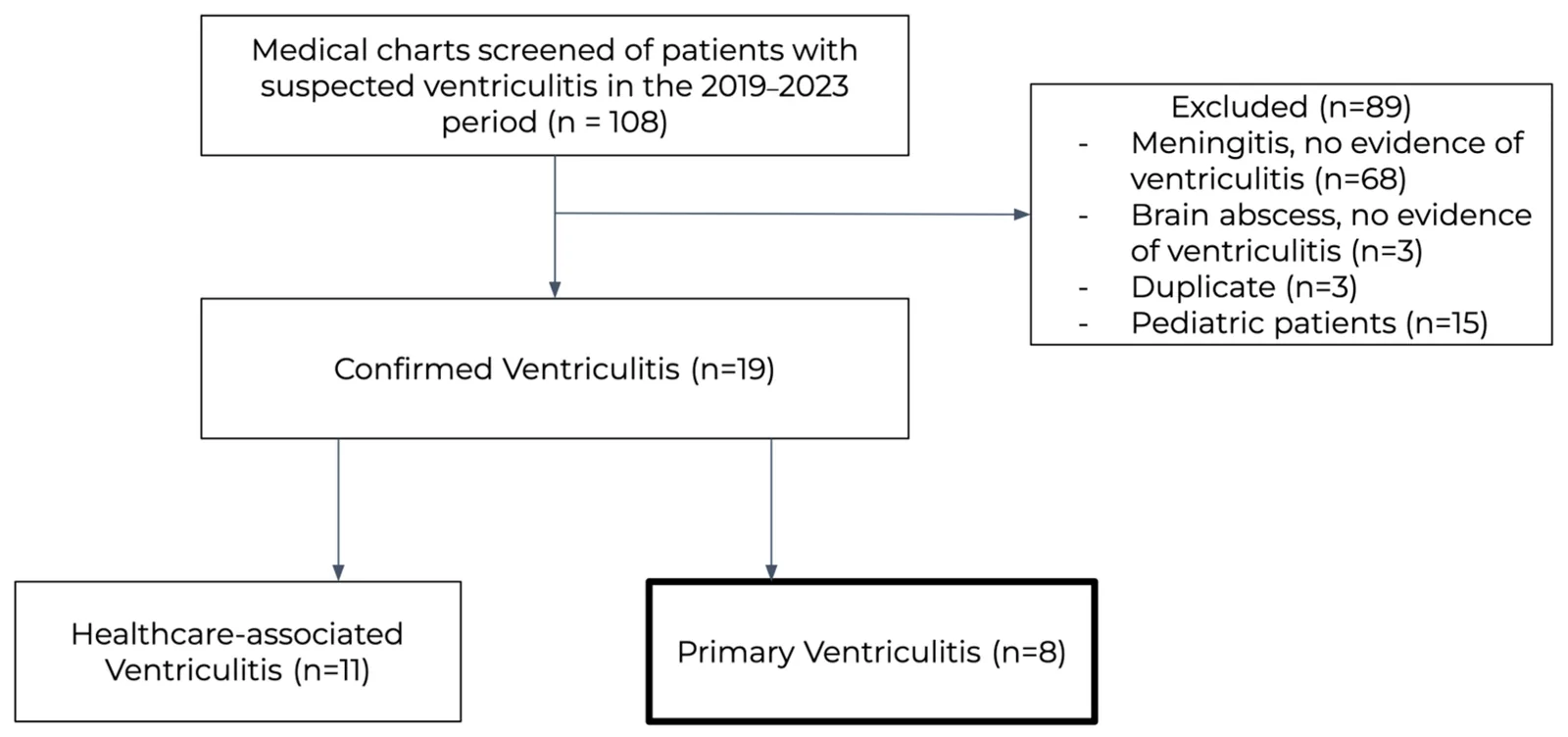
Patient selection and cohort definition flow diagram. Out of 108 screened medical charts, 89 cases were excluded based on strict exclusion criteria. Of the 19 confirmed ventriculitis cases, 11 were healthcare-associated and 8 met the criteria for primary ventriculitis (active study cohort).

During the 5-year study period (2019–2023), a total of *N* = 8 adult patients met the strict diagnostic criteria for primary pyogenic ventriculitis (Figure 1). As detailed in the unmasked master clinical table (Table 1), the overall cohort had a median age of 60.5 years (IQR: 51.5–66.5 years; range: 42–71 years), with a male predominance (62.5%, *n* = 5/8). Baseline comorbidities included diabetes mellitus (37.5%, *n* = 3/8), hypertension (37.5%, *n* = 3/8), chronic kidney disease (12.5%, *n* = 1/8), alcoholism (12.5%, *n* = 1/8), liver cirrhosis (12.5%, *n* = 1/8), and coronary artery disease (12.5%, *n* = 1/8). All patients presented with acute, severe neurological compromise. The median initial Glasgow Coma Scale (GCS) score at admission was 7.5 (IQR: 4.0–9.75; range: 3–12), with 75.0% (*n* = 6/8) presenting in severe coma (GCS ≤ 8). Three patients (37.5%; Pt #4, Pt #7, Pt #8) presented in refractory septic shock requiring immediate, continuous vasopressor support upon ICU admission.

### 3.2. CSF Parameters and Microbiological Yield

Across the total cohort (*N* = 8), initial CSF analysis demonstrated a median white blood cell (WBC) count of 687.0 cells/uL (IQR: 396.3–1317.5 cells/uL), a median protein concentration of 910.0 mg/dL (IQR: 663.0–1142.5 mg/dL), and a median glucose concentration of 37.0 mg/dL (IQR: 22.5–57.5 mg/dL).

Descriptive evaluation of individual cases revealed distinct cytochemical patterns based on 1-year clinical survival. Surviving patients (*n* = 6, Pt #1–#6) mounted a robust initial CSF cellular and protein inflammatory response, with a median WBC count of 1005.0 cells/uL (IQR: 508.0–1977.5 cells/uL) and a median protein level of 1030.0 mg/dL (IQR: 847.5–1225.0 mg/dL). CSF glucose was markedly depressed (median: 21.0 mg/dL; IQR: 15.8–39.5 mg/dL). In contrast, non-survivors (*n* = 2, Pt #7, Pt #8) displayed a blunted CSF cellularity and lower protein response at presentation. Pt #7 exhibited a CSF WBC count of 115 cells/uL and protein of 284.2 mg/dL; Pt #8 exhibited a CSF WBC count of 50 cells/uL and protein of 140.0 mg/dL (group median WBC: 82.5 cells/uL; protein: 212.1 mg/dL). CSF glucose in non-survivors was comparatively higher (Pt #7: 92.0 mg/dL; Pt #8: 83.0 mg/dL).

Microbiological cultures yielded positive pathogen identification in 25.0% (*n* = 2/8) of cases: *Streptococcus pneumoniae* was isolated from blood and CSF in Pt #2, and *Staphylococcus aureus* was cultured from CSF in Pt #5.

The remaining 6 cases (75.0%) yielded culture-negative CSF, predominantly reflecting empiric intravenous antimicrobial administration at primary referring hospitals prior to tertiary neurosurgical transfer.

Confounder analysis revealed that systemic blood glucose levels at presentation were elevated in patients with underlying diabetes mellitus (Pt #1: 215 mg/dL; Pt #4: 240 mg/dL; Pt #8: 285 mg/dL), directly influencing observed CSF-to-blood glucose ratios. Furthermore, CSF sampling sources varied: non-survivors (Pt #7, Pt #8) underwent immediate ventricular CSF sampling via emergency EVD placement, whereas survivors predominantly underwent initial diagnostic Lumbar Puncture (LP) (Table 1).

### 3.3. Neuroimaging Features

All 8 patients underwent cranial magnetic resonance imaging (MRI) including Diffusion-Weighted Imaging (DWI) and contrast-enhanced sequences upon clinical presentation.

**Figure 2:**
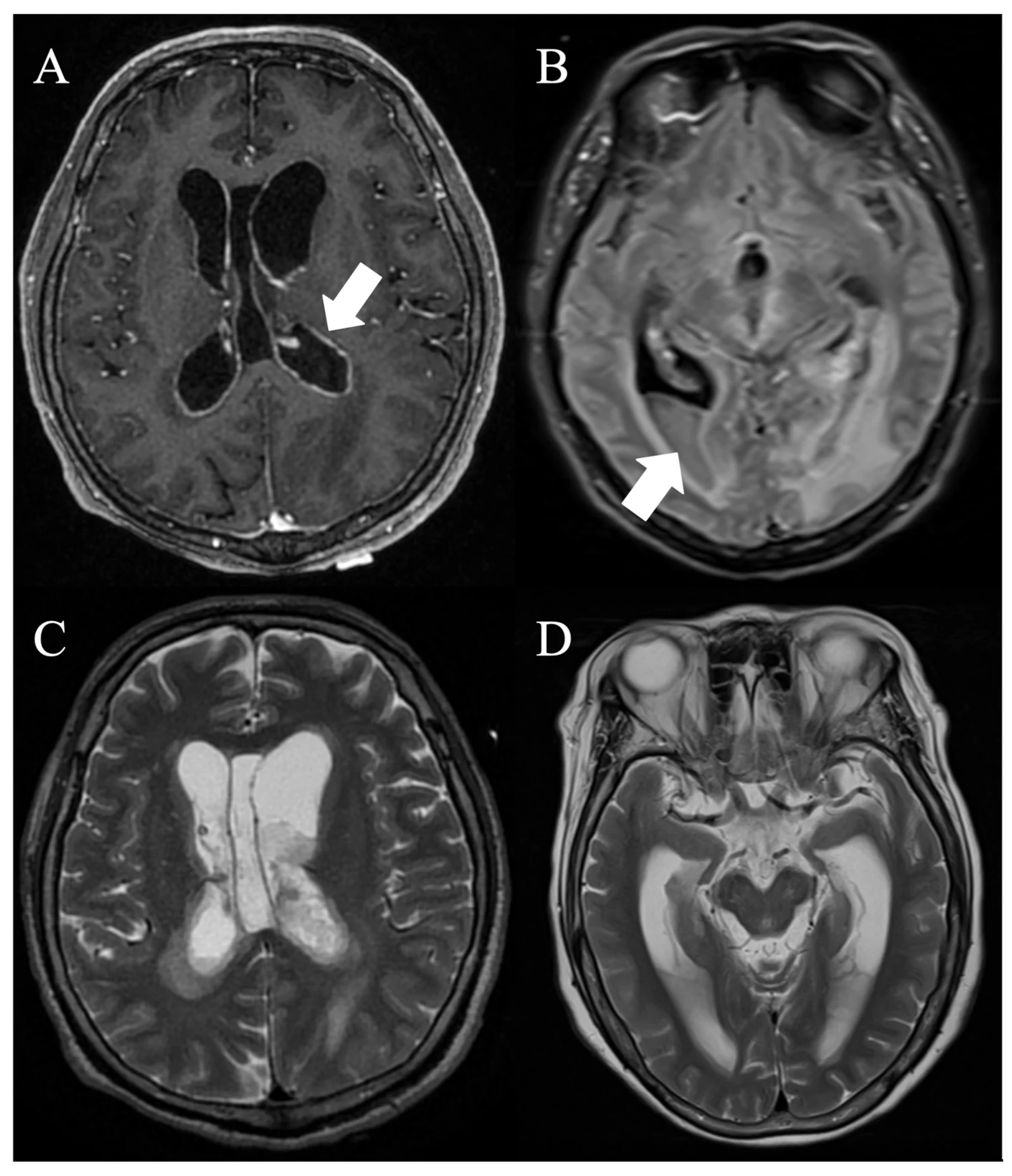
Representative cranial magnetic resonance imaging (MRI) features of a 1-year survivor (Pt #2) obtained on Day 3 post-symptom onset (**A**) Ependymal Contrast Enhancement: Axial contrast-enhanced T1-weighted sequence demonstrating diffuse, smooth, and continuous linear enhancement of the ependymal lining of the lateral ventricles (arrows). This indicates severe active inflammation of the ventricular wall and breakdown of the blood–cerebrospinal fluid barrier (BCSFB). (**B**) Intraventricular Purulent Debris (Pus): Axial T2-weighted/FLAIR sequence showing a distinct fluid-fluid level (arrows) in the dependent portion of the occipital horn of the lateral ventricle, representing the gravity-dependent layering of highly cellular, high-viscosity purulent exudate. (**C**) Intraventricular Debris and Periventricular Edema: Axial T2-weighted sequence demonstrating ventriculomegaly with layered, intermediate-signal intraventricular debris within the dependent portions of the lateral ventricles. Prominent surrounding T2 hyperintensity represents periventricular (peripheral) transependymal edema, indicative of acute cerebrospinal fluid accumulation and elevated intraventricular pressure. (**D**) Temporal Horn Dilatation: Axial T2-weighted sequence at the level of the midbrain, showing massive dilatation of the temporal horns of the lateral ventricles bilaterally. Prominent temporal horn enlargement is a highly sensitive and specific radiological marker of acute obstructive hydrocephalus requiring urgent neurosurgical CSF diversion.

Ependymal contrast enhancement along the ventricular walls was observed in 50.0% (*n* = 4/8) of cases (survivors: *n* = 3/6 [50.0%]; non-survivors: *n* = 1/2 [50.0%]). Intraventricular purulent debris displaying restricted diffusion on DWI was identified in 50.0% (*n* = 4/8) of patients (survivors: *n* = 3/6; non-survivors: *n* = 1/2), as illustrated in Figure 2. Additionally, acute obstructive hydrocephalus secondary to ventricular inflammatory exudates was documented in 50.0% (*n* = 4/8) of cases.

### 3.4. Clinical Outcomes and Surgical Interventions

Surgical management via External Ventricular Drain (EVD) placement was performed in 87.5% (*n* = 7/8) of patients to manage acute obstructive hydrocephalus and evacuate intraventricular debris; Pt #2 was successfully managed with serial therapeutic lumbar punctures alone. The timing of EVD placement reflected distinct clinical contexts across individual patients. Among surviving patients (*n* = 6), EVD placement occurred at a median of 10.5 days post-symptom onset (IQR: 3.8–12.5 days), typically following secondary development of progressive obstructive hydrocephalus or persistent purulent debris during intravenous antibiotic therapy. In contrast, non-survivors (*n* = 2) underwent emergency EVD placement significantly earlier, at a median of 2.5 days post-symptom onset (Pt #7 at Day 2; Pt #8 at Day 3). Both fatal cases presented in profound initial coma (GCS 4 and 3, respectively) with refractory septic shock, compelling urgent EVD placement as a reactive rescue maneuver for acute hydrocephalus (confounding by indication).

Overall 1-year mortality was 25.0% (*n* = 2/8, Pt #7 and Pt #8), with deaths occurring on hospitalization Days 18 and 9, respectively. Both fatal cases occurred in male patients presenting with severe coma (GCS ≤ 4), vasopressor-dependent septic shock, and blunted CSF cellularity.

Among the 6 surviving patients, the median 1-year modified Rankin Scale (mRS) score was 2.5 (IQR: 2.0–3.0; individual scores: Pt #1: mRS 2, Pt #2: mRS 1, Pt #3: mRS 3, Pt #4: mRS 4, Pt #5: mRS 2, Pt #6: mRS 3). Favorable to moderate functional recovery (mRS 1–3) was achieved in 83.3% (*n* = 5/6) of survivors.

**Figure 3:**
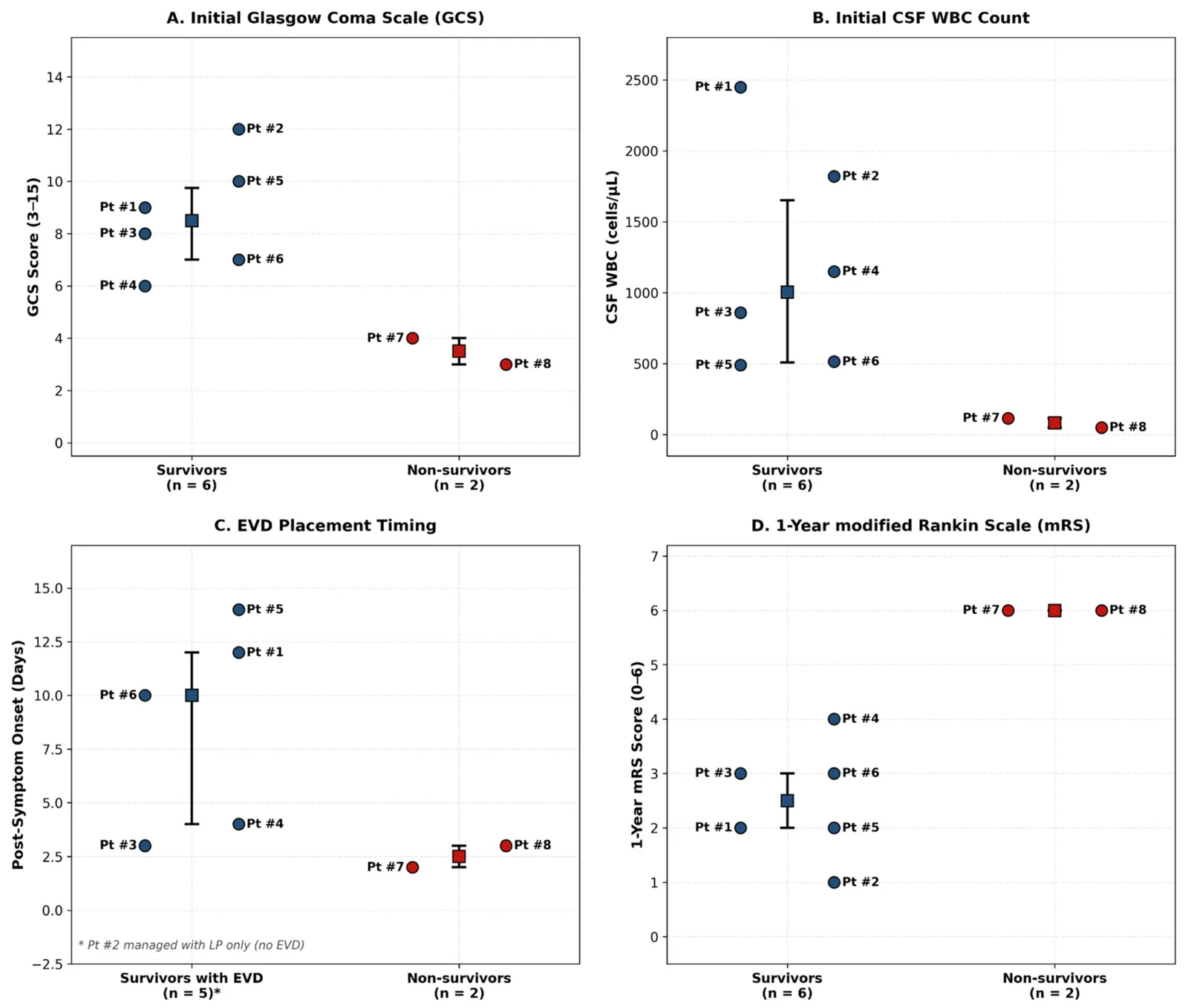
Individual patient-level distribution of clinical presentations, CSF parameters, surgical timelines, and 1-year functional outcomes in adult primary ventriculitis (*N* = 8). Blue circles represent individual survivors (*n* = 6); red circles represent individual non-survivors (*n* = 2). The solid blue and red squares with vertical error bars denote group medians and interquartile ranges (IQRs; 25th–75th percentiles) for survivors, and medians with absolute ranges (min–max) for non-survivors. Individual data points for admission GCS, initial CSF WBC count, CSF protein, CSF glucose, and EVD placement timing post-symptom onset are plotted across all cases to illustrate clinical heterogeneity between survivors (*n* = 6, Pt #1–#6) and non-survivors (*n* = 2, Pt #7, Pt #8). Early external ventricular drain (EVD) placement in non-survivors reflects a reactive rescue maneuver in severe initial coma (confounding by indication), while fatal cases exhibited a blunted initial CSF pleocytosis despite severe clinical presentation. In accordance with CARE reporting guidelines for rare case series, data are displayed as individual scatter distributions and non-parametric median descriptors without inferential hypothesis testing or *p*-value calculations.

Individual scatter distribution of GCS, CSF WBC, EVD timing, and outcomes across all *N* = 8 patients is depicted in Figure 3.

## 4. Discussion

### 4.1. Diagnostic Challenges, Neuroimaging Indications, and Molecular Diagnostics

Primary pyogenic ventriculitis in adults represents an exceptionally rare and diagnostically challenging central nervous system (CNS) emergency [3,6]. In our cohort, a definitive microbiological diagnosis via standard blood or CSF culture was achieved in only 25.0% (*n* = 2/8) of patients. The remaining six patients (75.0%) presented as a distinct culture-negative subgroup, primarily reflecting empiric broad-spectrum intravenous antimicrobial administration at primary referring hospitals prior to tertiary transfer. In this culture-negative subgroup, diagnostic confirmation relied on a synthesis of purulent CSF cytochemical parameters, classic clinical meningeal signs, and supportive neuroimaging findings, alongside the systematic exclusion of secondary or iatrogenic infectious sources. Compared with reported community-acquired CNS infection cohorts, where pre-referral antimicrobial exposure similarly blunts microbiological yield, primary pyogenic ventriculitis presents a pronounced diagnostic bottleneck distinct from post-neurosurgical series, where direct intraventricular catheter sampling typically yields higher culture positivity.

Advanced cranial magnetic resonance imaging (MRI) serves as a valuable non-invasive diagnostic tool when CSF cultures remain negative. High-resolution MRI utilizing Diffusion-Weighted Imaging (DWI) and contrast-enhanced T1-weighted sequences effectively delineates intraventricular exudates. Purulent ventricular debris, characterized by high cellularity and hyperviscosity, restricts the Brownian motion of water molecules, manifesting as hyperintensity on DWI with corresponding hypointensity on Apparent Diffusion Coefficient (ADC) maps. This restricted diffusion provides a sensitive imaging feature that helps distinguish pyogenic intraventricular empyema from sterile intracranial hemorrhage or CSF flow artifacts. In our series, intraventricular debris and ependymal contrast enhancement were documented in 50.0% (*n* = 4/8) of patients. Because its diagnostic sensitivity is variable, neuroimaging serves as a supportive diagnostic adjunct that must be interpreted in conjunction with acute CSF inflammatory trajectories and clinical presentation, rather than viewed as a standalone primary driver of therapeutic decision-making.

To overcome the limitations of culture-negative presentations, integrating molecular diagnostic techniques—specifically cerebrospinal fluid metagenomic Next-Generation Sequencing (mNGS) or multiplex polymerase chain reaction (PCR) panels—into early diagnostic algorithms holds substantial promise for rapid, unbiased pathogen identification without requiring live bacterial growth [1,11].

### 4.2. Physiological, Anatomical, and Pharmacological Confounders of CSF Inflammatory Profiles

Analysis of individual CSF cytochemical parameters in our cohort revealed marked descriptive variations between surviving and non-surviving patients. Surviving patients (*n* = 6) mounted a robust localized pleocytosis and protein elevation (median CSF WBC: 1005.0 cells/µL; median protein: 1030.0 mg/dL) accompanied by severe hypoglycorrhachia (median CSF glucose: 21.0 mg/dL). In contrast, fatal cases (*n* = 2; Pt #7 and Pt #8) presented with blunted CSF pleocytosis (WBC: 115.0 and 50.0 cells/µL) and lower protein concentrations (284.2 and 140.0 mg/dL) despite catastrophic clinical initial presentations [6,12].

Crucially, interpreting these observed CSF cytochemical variations requires a comprehensive evaluation of several major physiological, anatomical, and pharmacological confounders. First, age differences across the cohort—with non-survivors being markedly older (median age: 65.5 vs. 53.3 years in survivors)—indicate that age-related immunosenescence and altered blood-CSF barrier function may attenuate acute localized leukocyte recruitment. Second, paired admission laboratory parameters demonstrate that the higher CSF glucose observed in non-survivors (median: 87.5 vs. 30.0 mg/dL in survivors) was directly driven by severe systemic hyperglycemia in patients with underlying diabetes mellitus (admission serum blood glucose up to 285.0 mg/dL). Because glucose passively diffuses across the blood-CSF barrier down a concentration gradient, systemic hyperglycemia artificially elevates absolute CSF glucose levels, effectively masking underlying severe hypoglycorrhachia. Third, peripheral leukocyte profiles and pre-referral antimicrobial exposure profoundly modulated central inflammatory findings; fatal cases presented in refractory septic shock with severe peripheral leukopenia (e.g., Pt #8 peripheral WBC count: 3100/µL), while empiric broad-spectrum antibiotic administration at primary referring hospitals prior to tertiary transfer suppressed bacterial viability and blunted cellular recruitment across 75.0% of the cohort. Finally, anatomical CSF sampling sources differed fundamentally across subgroups: non-survivors underwent immediate ventricular CSF drainage via emergency EVD placement, whereas survivors predominantly underwent initial diagnostic lumbar puncture. Rostrocaudal concentration gradients of CSF protein and cellular elements between the lumbar cistern and cerebral ventricles introduce inherent analytical variability that must be considered when evaluating diagnostic trajectories. While systemic immunoparalysis secondary to severe septic shock remains a plausible hypothesis, future prospective investigations incorporating immunophenotyping, cytokine panels, and serial paired serum-CSF analyses are required to elucidate these mechanisms [1,12].

### 4.3. Pharmacokinetic BBB/BCSFB Barriers and Targeted Intraventricular Pharmacotherapy

The central therapeutic bottleneck in primary ventriculitis is governed by the structural integrity of the blood–brain barrier (BBB) and blood–cerebrospinal fluid barrier (BCSFB). Complex tight junctions between cerebrovascular endothelial cells and choroid plexus epithelial cells severely restrict the passive diffusion of hydrophilic, high-molecular-weight systemically administered antimicrobials—including vancomycin, aminoglycosides, and colistin—preventing them from achieving minimum inhibitory concentrations (MIC) within the purulent ventricular lumen [7].

To circumvent these pharmacokinetic constraints, current Infectious Diseases Society of America (IDSA) guidelines recommend considering direct intraventricular (IVT) or intrathecal (IT) antimicrobial administration in refractory cases or infections caused by multidrug-resistant pathogens [1]. Direct IVT instillation achieves immediate, supratherapeutic antimicrobial concentrations localized within the ventricular system while minimizing systemic organ toxicity. Meta-analytical evidence by Florez-Perdomo et al. (2023) demonstrated that adjunctive IVT/IT therapy combined with systemic administration significantly reduces overall mortality (OR: 0.27, 95% CI: 0.15–0.49) and enhances microbiological cure rates (OR: 3.4, 95% CI: 1.6–7.22) in complex ventriculitis [13]. When IVT antimicrobials are instilled via an EVD, clamping the drain for 15 to 60 min is essential to allow uniform pharmacokinetic distribution throughout the ventricular cerebrospinal fluid pathways prior to resuming therapeutic drainage [1].

### 4.4. Surgical Source Control: EVD Timing, Indication Bias, and Emerging Irrigation Modalities

In our cohort, surgical intervention via EVD placement was performed in 87.5% (*n* = 7/8) of patients. Non-survivors underwent EVD placement significantly earlier post-symptom onset (median: 2.5 days; Pt #7 at Day 2, Pt #8 at Day 3) compared to survivors (median: 10.5 days; IQR: 3.8–12.5 days). This timing discrepancy represents a classic confounding by indication (indication bias) [3,6]. Both fatal cases presented in profound initial coma (GCS 4 and 3) accompanied by vasopressor-dependent septic shock, compelling urgent EVD insertion as a reactive rescue maneuver for acute obstructive hydrocephalus and severe intracranial hypertension. Conversely, EVD placement in surviving patients was typically delayed until secondary obstructive hydrocephalus or persistent purulent debris developed during ongoing intravenous antibiotic therapy.

Passive EVD placement alone may be insufficient in severe ventriculitis, as highly viscous, fibrin-rich intraventricular exudates frequently cause catheter occlusion, intraventricular loculation, and ependymal scarring [6,12]. Emerging surgical source control strategies, such as Neuroendoscopic Lavage (NEL) or continuous active CSF exchange catheter systems (e.g., IRRAflow), offer innovative avenues for physical debridement [14,15]. By directly aspirating dense purulent empyema, fenestrating loculated inflammatory septations, and performing extensive warm saline irrigation under direct visualization, active surgical debridement physically debulks bacterial load and inflammatory cytokines, potentially preventing permanent shunt dependency [14,15]. However, because existing evidence regarding catheter-associated biofilm prevention and ependymal scarring is predominantly derived from nosocomial device-related ventriculitis literature, direct extrapolation to spontaneous primary pyogenic ventriculitis must be interpreted with caution. In non-device-related primary ventriculitis, early surgical decompression serves primarily as an adjunct for intracranial pressure control and exudate clearance, rather than an established disease-modifying intervention against biofilm dynamics.

While no patients in our retrospective series underwent active lavage, future prospective trials are warranted to evaluate whether early surgical irrigation improves survival over passive EVD.

### 4.5. Neuroendoscopic Lavage and Active CSF Exchange for Source Control

While passive external ventricular drainage (EVD) placement represents the traditional baseline of surgical care, standard ventricular catheters remain highly susceptible to mechanical occlusion when draining thick, viscous, and fibrinous intraventricular empyema [6,12]. To address this mechanical constraint, recent neurosurgical literature has explored a transition toward active debridement strategies, specifically neuroendoscopic lavage (NEL) and automated active closed-loop cerebrospinal fluid exchange (ACE) systems [14,15].

Neuroendoscopic lavage (NEL), performed under direct high-definition visualization, enables surgeons to aspirate dense purulent debris, fenestrate inflammatory septations to reconnect isolated ventricular compartments, and perform copious warm saline irrigation [14,15]. Observational series suggest that physical debulking via NEL reduces local bacterial burden and inflammatory cytokine concentrations, thereby enhancing local antimicrobial efficacy and facilitating the clearance of refractory intraventricular suppuration. Furthermore, this active clearance has been associated with reduced rates of permanent shunt dependency and long-term cognitive deficits [15]. For instance, a systematic synthesis by Basit et al. (2025) [14] noted accelerated CSF normalization following ventricular lavage compared to conservative therapy, with median WBC clearance improving from 12.0 to 4.0 days and protein normalization from 27.2 to 12.9 days. Complementing intraoperative intervention, automated active closed-loop CSF exchange (ACE) catheter systems (e.g., IRRAflow) combine continuous automated irrigation with active fluid clearance and integrated real-time ICP monitoring to mitigate catheter occlusion [16,17]. In a recent multicenter retrospective cohort study by Rezai Jahromi et al. (2025), ACE therapy was associated with reduced drainage failure (OR: 0.21, 95% CI: 0.08–0.54), lower secondary catheter-associated infection rates (OR: 0.20, 95% CI: 0.06–0.62), decreased shunt dependency (OR: 0.28, 95% CI: 0.11–0.71), and an increased likelihood of achieving favorable 1-year functional recovery (mRS ≤ 2) [18].

Crucially, the current evidence base supporting both NEL and active fluid-exchange systems consists exclusively of small retrospective cohorts, non-randomized observational comparative studies, and case series, representing Oxford Class III/Level 4 evidence [14,17,18]. Furthermore, these clinical series have been conducted predominantly in healthcare-associated, post-neurosurgical, or indwelling device-related infection cohorts rather than spontaneous primary pyogenic ventriculitis. Consequently, all reported treatment metrics and observational odds ratios (OR) must be interpreted with substantial caution, as they are inherently susceptible to selection bias, publication bias, and confounding by indication (where surgical debridement is preferentially offered to candidates with more favorable baseline physiological reserve). While active surgical debridement represents a promising surgical avenue, prospective randomized controlled trials (RCTs) are strictly required to validate its therapeutic efficacy in primary ventriculitis.

## 5. Limitations

Several methodological and clinical limitations inherent to this study must be acknowledged in accordance with CARE reporting guidelines [19]. First, primary pyogenic ventriculitis in adults remains an exceptionally rare neuro-infectious emergency; consequently, our single-center cohort of eight patients accumulated over a 5-year period precludes inferential statistical hypothesis testing and multivariable risk adjustment. To prevent false-positive inferential claims and type I errors, descriptive metrics (Median and Interquartile Range) were intentionally prioritized [6,19]. Second, the retrospective design utilizing electronic health records introduces potential selection bias, unmeasured residual confounding, and an absence of granular long-term neurocognitive or fatigue assessments. Third, substantial diagnostic heterogeneity was introduced by high pre-referral antimicrobial exposure, which resulted in a 75.0% culture-negativity rate and necessitated reliance on clinical criteria and non-invasive Diffusion MRI features [3,6]. Finally, because existing management benchmarks are predominantly derived from Western healthcare systems, transferability to distinct regional referral logistics requires cautious interpretation. To address these constraints, future prospective, multicenter studies incorporating standardized molecular diagnostics (mNGS), therapeutic drug monitoring for intraventricular antimicrobials, and randomized evaluations of timely surgical irrigation modalities are urgently needed to optimize survival and functional recovery [1,11,14].

## 6. Conclusions

Primary pyogenic ventriculitis in adults is a rare, life-threatening central nervous system emergency characterized by marked diagnostic ambiguity and high clinical severity. In our retrospective cohort, conventional microbiological culture yield was severely constrained (25.0%, *n* = 2/8), primarily driven by pre-referral antimicrobial exposure. In culture-negative presentations, high-resolution cranial MRI demonstrating intraventricular restricted diffusion on DWI/ADC maps provides a valuable, non-invasive imaging feature to support diagnostic evaluation and guide prompt clinical decision-making.

Furthermore, our study demonstrates that earlier emergency EVD placement in fatal cases was compelled by severe initial presentation—specifically profound coma (GCS 3–4) and refractory septic shock—reflecting a reactive rescue maneuver for acute intracranial hypertension rather than an inherent driver of poor prognosis (confounding by indication). Timely neuroimaging recognition using Diffusion MRI, coupled with prompt antimicrobial coverage and appropriate surgical source control, remains paramount for optimizing clinical decision-making and functional outcomes in this critical condition.

## Figures and Tables

**Table 1:** Demographics, baseline characteristics, cerebrospinal fluid parameters, surgical management, and 1-year clinical outcomes across individual patients (*N* = 8).

Patient ID	Age/Sex	Baseline Comorbidities	Systemic Blood Glucose (mg/dL)	CSF Sampling Source & Day	Initial CSF WBC (cells/µL)	Initial CSF Protein (mg/dL)	Initial CSF Glucose (mg/dL)	Microbiological Etiology	MRI Features (DWI/Ependymal)	Initial GCS	EVD Placement (Day Post-Onset)	Vasopressor Support	1-Year Survival	1-Year mRS
Pt #1	62/M	DM, HTN	215	LP (Day 2)	2450	1280.0	18.0	Culture-negative	Debris (+), Enhancement (+)	9	Day 12	No	Survived	2
Pt #2	48/F	None	105	LP (Day 1)	1820	940.0	24.0	*Streptococcus pneumoniae*	Debris (+), Enhancement (+)	12	None (LP only)	No	Survived	1
Pt #3	55/M	HTN	128	EVD (Day 3)	860	1120.0	32.0	Culture-negative	Debris (+), Enhancement (+)	8	Day 3	No	Survived	3
Pt #4	71/F	DM, CKD	240	EVD (Day 4)	1150	880.0	42.0	Culture-negative	Debris (−), Enhancement (+)	6	Day 4	Yes	Survived	4
Pt #5	42/M	Alcoholism	112	LP (Day 1)	490	1210.0	15.0	*Staphylococcus aureus*	Debris (+), Enhancement (+)	10	Day 14	No	Survived	2
Pt #6	68/F	HTN	135	LP (Day 2)	514	749.3	49.0	Culture-negative	Debris (−), Enhancement (+)	7	Day 10	No	Survived	3
Pt #7	59/M	CAD	145	EVD (Day 2)	115	284.2	92.0	Culture-negative	Debris (+), Enhancement (+)	4	Day 2	Yes	Fatal	6 (Died Day 18)
Pt #8	66/M	DM, Liver Cirrhosis	285	EVD (Day 3)	50	140.0	83.0	Culture-negative	Debris (−), Enhancement (+)	3	Day 3	Yes	Fatal	6 (Died Day 9)

Abbreviations: CAD, coronary artery disease; CKD, chronic kidney disease; CSF, cerebrospinal fluid; DM, diabetes mellitus; EVD, external ventricular drain; GCS, Glasgow Coma Scale; HTN, hypertension; LP, lumbar puncture; mRS, modified Rankin Scale; WBC, white blood cell.

## Data Availability

The raw data supporting the conclusions of this article will be made available by the authors on request due to patient privacy and ethical restrictions.

## References

[1] Tunkel A.R., Hasbun R., Bhimraj A., Byers K., Kaplan S.L., Scheld W.M., van de Beek D., Bleck T.P., Garton H.J., Zunt J.R. (2017). 2017 Infectious Diseases Society of America’s Clinical Practice Guidelines for Healthcare-Associated Ventriculitis and Meningitis. Clin. Infect. Dis..

[2] Gronthoud F., Hassan I., Newton P. (2017). Primary pyogenic ventriculitis caused by Neisseria meningitidis: Case report and review of the literature. JMM Case Rep..

[3] Díez-Vidal A. (2025). Ventriculitis: A Retrospective Cohort Study of Clinical Features, Outcomes and Device Management Practices. Open Forum Infect. Dis..

[4] Adhikari P., Antala D., Pyakuryal B., Muhammed A., Pudasainee P., Friedman H., Ezepue C.J. (2022). Community-Acquired Meningitis Complicated by Pyogenic Ventriculitis: A Case Report. Cureus.

[5] Spector R., Keep R.F., Snodgrass R.S., Smith Q.R., Johanson C.E. (2015). A balanced view of choroid plexus structure and function: Focus on adult humans. Exp. Neurol..

[6] Luque-Paz D., Revest M., Eugène F., Boukthir S., Dejoies L., Tattevin P., Le Reste P.-J. (2021). Ventriculitis: A Severe Complication of Central Nervous System Infections. Open Forum Infect. Dis..

[7] Nau R., Sörgel F., Eiffert H. (2010). Penetration of drugs through the blood-cerebrospinal fluid/blood-brain barrier for treatment of central nervous system infections. Clin. Microbiol. Rev..

[8] Lesourd A., Magne N., Soares A., Lemaitre C., Taha M.-K., Gueit I., Wolff M., Caron F. (2018). Primary bacterial ventriculitis in adults, an emergent diagnosis challenge: Report of a meningoccal case and review of the literature. BMC Infect. Dis..

[9] Fukui M.B., Williams R.L., Mudigonda S. (2001). CT and MR imaging features of pyogenic ventriculitis. Am. J. Neuroradiol..

[10] Hong J.T., Son B.C., Sung J.H., Kim I.S., Yang S.H., Lee S.W., Park C.K. (2008). Significance of diffusion-weighted imaging and apparent diffusion coefficient maps for the evaluation of pyogenic ventriculitis. Clin. Neurol. Neurosurg..

[11] Wilson M.R., Sample H.A., Zorn K.C., Arevalo S., Yu G., Neuhaus J., Federman S., Stryke D., Briggs B., Langelier C. (2019). Clinical Metagenomic Sequencing for Diagnosis of Meningitis and Encephalitis. N. Engl. J. Med..

[12] Beer R., Lackner P., Pfausler B., Schmutzhard E. (2008). Nosocomial ventriculitis and meningitis in neurocritical care patients. J. Neurol..

[13] Florez-Perdomo W.A., Escobar-Cardona D., Janjua T., Agrawal A., Vasquez H., Lozada-Martinez I.D., Moscote-Salazar L.R. (2023). Antibiotic therapy by intrathecal or intraventricular approach for postsurgical meningitis or ventriculitis: A systematic review and meta-analysis. Egypt. J. Neurosurg..

[14] Basit A., Ahmed I., Waseem D., Shaikh S.S., Suleman F., Khan S.A., Hussain M., Urooj R., Qamar M.A., Akhunzada N.Z. (2025). Ventricular lavage versus conservative treatment in cerebral ventriculitis outcomes: A systematic review. Acta Neurol. Belg..

[15] Bal’afif F., Nazwar T.A., Wardhana D.W., Sanjaya O.T., Mataniari K.N., Saputri F.S., Dewi L., Arifani H., Triana N.N., Mustofa (2024). Neuroendoscopic Lavage in Hydrocephalus with Ventricular Empyema: A Case Report. Dubai Med. J..

[16] Hess R.M., Khan A., Edwards M., Siddiqui A.H., Levy E.I. (2021). Continuous intraventricular vancomycin for treatment of ventriculitis using IRRAflow^®^: A case report. Surg. Neurol. Int..

[17] Stati G., Migliorino E., Moneti M., Castioni C.A., Scibilia A., Palandri G., Virgili G., Aspide R. (2023). Treatment of cerebral ventriculitis with a new self-irrigating catheter system: Narrative review and case series. J. Anesth. Analg. Crit. Care.

[18] Rezai Jahromi B., Badr R., Asbury D., Unser J. (2025). Comprehensive Engineering Performance Evaluation of the IRRAflow Active Fluid Exchange System with IRRAS Bolt for Intracranial Fluid Management. Cureus.

[19] Gagnier J.J., Kienle G., Altman D.G., Moher D., Sox H., Riley D. (2013). The CARE Guidelines: Consensus-based Clinical Case Reporting Guideline Development. Glob. Adv. Health Med..

